# Binge Drinking Among Sports Gamblers

**DOI:** 10.1001/jamanetworkopen.2024.5473

**Published:** 2024-04-01

**Authors:** Joshua B. Grubbs, Shane W. Kraus

**Affiliations:** 1 Department of Psychology, Center on Alcohol, Substance use, And Addictions, University of New Mexico, Albuquerque; 2 Department of Psychology, University of Nevada, Las Vegas

## Abstract

This survey study examines whether or not individuals who wager on sports are at greater risk of binge use of alcohol.

## Introduction

Over the past 6 years, sports wagering has become accessible to most individuals in the US via mobile applications or websites.^1^ Increasing evidence suggests that sports wagering is associated with greater substance use and misuse, particularly alcohol, and symptoms of alcohol use disorder.^2,3,4^ Alcohol consumption is higher among sports gamblers,^3^ and sports gamblers often use substances while gambling.^5^ Sports gamblers tend to be more inclined toward risk taking, suggesting that sports gambling may be associated with more risky alcohol use behaviors.^4,5,6^ Accordingly, we examined whether individuals who wager on sports in the US are at greater risk of binge use of alcohol.

## Methods

This survey study was approved by the Bowling Green State University Institutional Review Board; informed consent was obtained from all participants. The study followed the AAPOR reporting guideline.

From March 17 to April 6, 2022, we collected a census-matched sample of US adults with an oversample of adults who wager on sports. Full information about this survey is available elsewhere.^1^ Race and ethnicity data were collected because they are potential factors in sports gambling likelihood and binge drinking habits. Among those reporting any past year alcohol use, binge drinking was assessed via the National Institute on Drug Abuse Quick Screen, version 1.0, which asks how often respondents consumed an excess of alcohol at a single time (≥5 drinks for men; ≥4 for women). Participants responded on a scale of 1 (never) to 5 (daily or more). Sports betting status was assessed by asking participants whether they had placed bets on sporting events or esports or participated in daily fantasy sports over the past 12 months.

Statistical analyses were conducted in SPSS, version 28. We conducted 2-tailed χ^2^ analyses for distributions of past year binge drinking frequency, followed by multinomial logistic regressions estimating binge drinking frequency; *P* < .05 was considered statistically significant.

## Results

A total of 4363 respondents were included (51.4% men, 46.4% women, and 2.2% nonbinary or other; mean [SD] age, 49.6 [16.2] years) (Table 1). The national census-matched survey consisted of 2806 participants (mean [SD] age, 48.9 [17.2] years; 1365 [48.6%] men and 1441 [51.4%] women; response rate, 2806 of 3203 [87.6%]). The oversample of sports gamblers consisted of 1557 participants (mean [SD] age, 41.7 [15.3] years; 1043 [67.0%] men and 514 [33.0%] women; response rate, 1557 of 1978 [78.7%]), of whom 1474 reported past year sports betting. Additionally, in the national sample, 338 respondents (12.0%) indicated they had gambled on sports in the past 12 months, resulting in a total of 1812 sports gamblers (Table 1). Sports gamblers were disproportionally likely to be men and younger. In these combined samples, 3267 respondents (74.9%) reported past year alcohol use.

**Table 1.** Demographic Characteristics of Total Sample and Gambling Groups^a^

Characteristic	Total (N = 4363)	Nongambler (n = 1326)^b^	Non–sports gambler (n = 1225)^b^	Sports gambler (n = 1812)^b^	Statistical analysis	*P* value
Age, mean (SD), y	49.6 (16.2)	50.1 (16.9)	54.5 (15.5)	45.8 (15.1)	*F*_2, 4360_ = 110.6	<.001
Gender						
Men	2243 (51.4)	535 (40.3) [*r_i_* = −5.6]	556 (45.4) [*r_i_* = −2.9]	1152 (63.6) [*r_i_* = 7.2]	χ^2^_6_ = 240.44	<.001
Women	2026 (46.4)	770 (58.1) [*r_i_* = 6.2]	657 (53.6) [*r_i_* = 3.7]	599 (33.1) [*r_i_* = −8.4]		
Nonbinary	60 (1.4)	17 (1.3) [*r_i_* = −0.3]	8 (0.7) [*r_i_* = −2.2]	35 (1.9) [*r_i_* = 2.0]		
Other^c^	34 (0.8)	4 (0.3) [*r_i_* = −2.0]	4 (0.3) [*r_i_* = −1.8]	26 (1.4) [*r_i_* = 3.2]		
Race and ethnicity						
American Indian or Alaska Native	71 (1.6)	9 (0.7) [*r_i_* = −2.7]	15 (1.2) [*r_i_* = −1.1]	47 (2.6) [*r_i_* = 3.2]	χ^2^_8_ = 58.84	<.001
Asian	140 (3.2)	51 (3.8) [*r_i_* = 1.3]	24 (2.0) [*r_i_* = −2.4]	65 (3.6) [*r_i_* = 0.9]		
Black	525 (12.0)	173 (13.0) [*r_i_* = 1.1]	136 (11.1) [*r_i_* = −0.9]	216 (11.9) [*r_i_* = −0.1]		
Hispanic	494 (11.3)	172 (13.0) [*r_i_* = 1.8]	152 (12.4) [*r_i_* = 1.1]	170 (9.4) [*r_i_* = −2.5]		
White	2911 (66.7)	850 (64.1) [*r_i_* = −1.2]	839 (68.5) [*r_i_* = 0.8]	1222 (67.4) [*r_i_* = 0.4]		
Multiple	104 (2.4)	36 (2.7) [*r_i_* = 0.8]	30 (2.4) [*r_i_* = 0.1]	38 (2.1) [*r_i_* = −0.8]		
Other^d^	118 (2.7)	35 (2.6) [*r*_i_ = −0.1]	29 (2.4) [*r*_i_ = −0.7]	54 (3.0) [*r*_i_ = 0.7]		
Binge frequency						
Men (n = 1712)^e^						
Never	614 (35.9)	166 (53.0) [*r_i_* = 5.1]	188 (43.0) [*r_i_* = 2.5]	260 (27.0) [*r_i_* = −4.6]	χ^2^_8_ = 110.57	<.001
Once or twice	483 (28.2)	84 (26.8) [*r_i_* = −0.5]	128 (29.3) [*r_i_* = 0.4]	271 (28.2) [*r_i_* = −0.02]		
Monthly	262 (15.3)	36 (11.5) [*r_i_* = −1.7]	52 (11.9) [*r_i_* = −1.8]	174 (18.1) [*r_i_* = 2.2]		
Weekly	254 (14.8)	22 (7.0) [*r_i_* = −3.6]	53 (12.1) [*r_i_* = −1.5]	179 (18.6) [*r_i_* = 3.0]		
Daily or almost daily	99 (5.8)	5 (1.6) [*r_i_* = −3.1]	16 (3.7) [*r_i_* = −1.8]	78 (8.1) [*r_i_* = 3.0]		
Women (n = 1555)^f^						
Never	680 (43.7)	275 (56.9) [*r_i_* = 4.4]	254 (50.9) [*r_i_* = 2.4]	151 (26.4) [*r_i_* = −6.3]	χ^2^_8_ = 154.75	<.001
Once or twice	442 (28.4)	127 (26.3) [*r_i_* = −0.9]	140 (28.1) [*r_i_* = 2.0]	175 (30.5) [*r_i_* = 1.0]		
Monthly	215 (13.8)	49 (10.1) [*r_i_* = −2.2]	57 (11.4) [*r_i_* = −1.4]	109 (19.0) [*r_i_* = 3.3]		
Weekly	148 (9.5)	26 (5.4) [*r_i_* = −2.9]	34 (6.8) [*r_i_* = −2.0]	88 (15.4) [*r_i_* = 4.5]		
Daily or almost daily	70 (4.5)	6 (1.2) [*r_i_* = −3.4]	14 (2.8) [*r_i_* = −1.8]	50 (8.7) [*r_i_* = 4.8]		

^a^ Unless specified otherwise, data are presented as No. (%) of participants.

^b^ For standardized residuals (*r_i_*) greater absolute values indicate greater deviation from expected count, with the sign of the residual indicating direction of the deviation so that negative values correspond to lower-than-expected frequencies and positive values correspond to higher-than-expected frequencies.

^c^ Participants could indicate this option if they did not identify as a man, woman, or nonbinary.

^d^ Includes Middle Eastern (owing to small sample size) and other races and ethnicities that were not specified.

^e^ Indicates 5 or more drinks at a single time.

^f^ Indicates 4 or more drinks at a single time.

Sports wagerers were disproportionately more likely to report binge drinking at monthly or greater frequency over the past 12 months and were also disproportionately less likely to report no binge drinking episodes in the past 12 months (Table 1). Multinomial logistic regressions adjusted for age and race and ethnicity showed that sports gamblers were substantially more likely to report higher levels of binge drinking (Table 2), suggesting that elevated risky drinking episodes among sports gamblers are not due to demographic differences.

**Table 2.** Multinomial Logistic Regression Estimating Binge Drinking Frequency in Past 12 Months

Characteristic	Gambling frequency, OR (95% CI)				*P* value
	Once or twice	Monthly	Weekly	Daily or almost daily	
**Women**					
Age	0.971 (0.963-0.979)	0.955 (0.944-0.965)	0.961 (0.949-0.974)	0.945 (0.926-0.963)	<.001
Race and ethnicity					
Asian	0.874 (0.435-1.757)	0.62 (0.247-1.561)	0.445 (0.124-1.589)	0.307 (0.039-2.45)	.052
American Indian or Alaska Native	0.609 (0.108-3.441)	1.495 (0.307-7.278)	5.97 (1.555-22.922)	5.22 (0.974-27.974)	
Black	1.021 (0.684-1.522)	0.895 (0.529-1.512)	1.488 (0.863-2.564)	2.715 (1.395-5.281)	
Hispanic	1.350 (0.924-1.972)	0.947 (0.568-1.578)	1.07 (0.586-1.953)	1.183 (0.51-2.743)	
White	1 [Reference]	1 [Reference]	1 [Reference]	1 [Reference]	
Multiple	0.665 (0.305-1.449)	0.506 (0.179-1.433)	1.21 (0.468-3.125)	0.756 (0.16-3.579)	
Other^a^	1.014 (0.464-2.218)	1.336 (0.549-3.252)	1.189 (0.404-3.503)	2.574 (0.818-8.100)	
Type of gambler					
Nongambler	1 [Reference]	1 [Reference]	1 [Reference]	1 [Reference]	<.001
Sports gambler	2.416 (1.773-3.291)	3.829 (2.562-5.724)	5.854 (3.586-9.557)	14.379 (5.954-34.723)	
Non–sports gambler	1.376 (1.014-1.865)	1.583 (1.026-2.441)	1.767 (1.016-3.07)	3.584 (1.331-9.647)	
**Men**					
Age	0.977 (0.969-0.985)	0.969 (0.959-0.979)	0.968 (0.958-0.978)	0.951 (0.936-0.966)	<.001
Race and ethnicity					
American Indian or Alaska Native	1.665 (0.525-5.282)	0.702 (0.130-3.800)	1.673 (0.453-6.177)	2.549 (0.547-11.889)	.49
Asian	0.915 (0.448-1.871)	0.687 (0.273-1.728)	0.997 (0.433-2.297)	0.53 (0.116-2.429)	
Black	0.824 (0.532-1.276)	0.822 (0.478-1.414)	0.938 (0.555-1.586)	1.612 (0.823-3.161)	
Hispanic	1.223 (0.794-1.884)	1.629 (1.003-2.646)	1.072 (0.623-1.846)	1.686 (0.827-3.438)	
White	1 [Reference]	1 [Reference]	1 [Reference]	1 [Reference]	
Multiple	0.636 (0.274-1.474)	0.943 (0.37-2.402)	0.548 (0.175-1.717)	1.566 (0.474-5.172)	
Other^a^	0.926 (0.427-2.010)	0.579 (0.186-1.805)	0.288 (0.064-1.302)	2.072 (0.681-6.307)	
Type of gambler					
Nongambler	1 [Reference]	1 [Reference]	1 [Reference]	1 [Reference]	<.001
Sports gambler	1.911 (1.391-2.626)	2.868 (1.893-4.346)	4.727 (2.897-7.712)	8.85 (3.48-22.507)	
Non–sports gambler	1.469 (1.032-2.089)	1.442 (0.889-2.337)	2.431 (1.406-4.205)	3.394 (1.203-9.575)	

Abbreviation: OR, odds ratio.

^a^ Includes Middle Eastern (owing to small sample size) and other races and ethnicities that were not specified.

## Discussion

In this survey study, binge drinking in both men and women was reported at greater frequency among sports wagering individuals compared with nongamblers and non–sports gamblers. This study is limited by its cross-sectional design and use of nonprobability polling methods. Regardless, with past research showing that sports gamblers are more likely to report symptoms of alcohol use disorder, our results suggest that individuals who wager on sports use alcohol in particularly risky ways. Given the rapid spread of sports wagering in the US over recent years, this finding highlights an immense need for ongoing research, particularly to examine how novel gambling technologies influence the prevalence, presentation, and prevention of alcohol use disorders and related harms.

## Notes

Supplement.
Data Sharing Statement
