# Supplementary material for: Binge Drinking Among Sports Gamblers
Source: JAMA Netw Open. 2024 Apr 1;7(4):e245473. doi: 10.1001/jamanetworkopen.2024.5473 (PMC10985549; doi:10.1001/jamanetworkopen.2024.5473)
Supplement: Supplement. — Data Sharing Statement [file jamanetwopen-e245473-s001.pdf]

Data Sharing Statement

Grubbs. Binge Drinking Among Sports Gamblers. *JAMA Netw Open*. Published April 01, 2024.
doi:10.1001/jamanetworkopen.2024.5473

Data
Data available: Yes
Data types: Deidentified participant data
How to access data: Via Open Science Framework
When available: With publication

Supporting Documents
Document types: Statistical/analytic code
How to access documents: https://osf.io/hmdwc/
When available: With publication

Additional Information
Who can access the data: Publicly Available Via the Open Science Framework
https://osf.io/hmdwc/
Types of analyses: For any purpose
Mechanisms of data availability: Via Open Science Framework
